# A chemical link between methylamine and methylene imine and implications for interstellar glycine formation

**DOI:** 10.1038/s42004-022-00677-5

**Published:** 2022-05-12

**Authors:** Prasad Ramesh Joshi, Yuan-Pern Lee

**Affiliations:** 1 Department of Applied Chemistry and Institute of Molecular Science, National Yang Ming Chiao Tung University, Hsinchu, Taiwan; 2 Center for Emergent Functional Matter Science, National Yang Ming Chiao Tung University, Hsinchu, Taiwan; 3 Institute of Atomic and Molecular Sciences, Academia Sinica, Taipei, Taiwan

**Keywords:** Infrared spectroscopy, Origin of life, Reaction mechanisms

## Abstract

Methylamine CH_3_NH_2_ is considered to be an important precursor of interstellar amino acid because hydrogen abstraction might lead to the aminomethyl radical •CH_2_NH_2_ that can react with •HOCO to form glycine, but direct evidence of the formation and spectral identification of •CH_2_NH_2_ remains unreported. We performed the reaction H + CH_3_NH_2_ in solid *p*-H_2_ at 3.2 K and observed IR spectra of •CH_2_NH_2_ and CH_2_NH upon irradiation and when the matrix was maintained in darkness. Previously unidentified IR spectrum of •CH_2_NH_2_ clearly indicates that •CH_2_NH_2_ can be formed from the reaction H + CH_3_NH_2_ in dark interstellar clouds. The observed dual-cycle mechanism containing two consecutive H-abstraction and two H-addition steps chemically connects CH_3_NH_2_ and CH_2_NH in interstellar media and explains their quasi-equilibrium. Experiments on CD_3_NH_2_ produced CD_2_HNH_2_, in addition to •CD_2_NH_2_ and CD_2_NH, confirming the occurrence of H addition to •CD_2_NH_2_.

## Abstract (web summary)

The detection of glycine in comets is suggestive of a cosmic origin for amino acids on Earth, but the formation of glycine in interstellar media remains poorly understood. Here, infrared spectroscopy shows that the CH_2_NH_2_ radical can be formed from H + CH_3_NH_2_ in conditions relevant to dark interstellar clouds, supporting the proposal that glycine could be formed from the reaction of CH_2_NH_2_ and HOCO radicals.

## Introduction

For the origin of life on earth it has long been presumed that prebiotic molecules were delivered from interstellar space through meteorites or comets or asteroids^1^. The simplest amino acid, glycine NH_2_CH_2_C(O)OH, is a key building block of proteins. Both glycine and methylamine, CH_3_NH_2_, were detected in comets Wild 2 and 67P/Churyumov-Gerasimenko (67P/C-G); these observations provided strong evidence for a cosmic origin of amino acids on Earth^2,3^, but the nature of the formation of glycine and other prebiotic molecules during the star-forming process remains unclear.

Several paths have been proposed for the formation of glycine according to theoretical calculations. Among them, the radical–radical reactions

1 $$\bullet {{{{{{\rm{CH}}}}}}}_{2}{{{{{{\rm{NH}}}}}}}_{2}+\bullet {{{{{\rm{HOCO}}}}}}\to {{{{{{\rm{NH}}}}}}}_{2}{{{{{{\rm{CH}}}}}}}_{2}{{{{{\rm{C}}}}}}({{{{{\rm{O}}}}}}){{{{{\rm{OH}}}}}}$$

2 $$\bullet {{{{{{\rm{NH}}}}}}}_{2}+\bullet {{{{{{\rm{CH}}}}}}}_{2}{{{{{\rm{C}}}}}}({{{{{\rm{O}}}}}}){{{{{\rm{OH}}}}}}\to {{{{{{\rm{NH}}}}}}}_{2}{{{{{{\rm{CH}}}}}}}_{2}{{{{{\rm{C}}}}}}({{{{{\rm{O}}}}}}){{{{{\rm{OH}}}}}}$$

appear to be more important than the ionic channels^4^. Garrod^5^ and Suzuki et al^6^. employed a model consisting of processes in the gaseous phase, on grain surface, and in bulk ice in hot cores to indicate the key roles of reactions (1) and (2) in the formation of glycine on the grains. Sato et al. employed state-of-the-art DFT calculations and reported that reaction (1) is the most feasible route^7^; these authors proposed that the aminomethyl radical, •CH_2_NH_2_, might be produced from successive hydrogenation of HCN or H abstraction from CH_3_NH_2_ by •OH or •NH_2_^5–9^. A similar theoretical chemical model involving NH_3_ and •HOCO was also proposed to understand the main routes for the formation and decomposition of CH_3_NH_2_^10^.

Laboratory investigations to produce glycine from smaller precursors mimicking interstellar conditions have been extensive. UV photolysis and electron bombardment of various interstellar ice analogues such as CO/NH_3_/H_2_O or H_2_O/CH_3_NH_2_/CO_2_ at low temperatures were demonstrated to yield glycine^11–18^. Recently, Ioppolo et al.^19^ observed glycine formation from ices containing CH_3_NH_2_, CO, O_2_, and atomic H under conditions similar to dark interstellar clouds; this result indicates that glycine can be formed with no need for energetic irradiation such as UV photons or cosmic rays, that is, during a much earlier star-formation stage than previously assumed. These authors proposed that glycine was produced via a barrierless radical-radical surface reaction (1), in which •CH_2_NH_2_ was produced through H abstraction from CH_3_NH_2_ by •OH (produced from H + O_2_) or H atom and •HOCO was produced via reaction between •OH and CO.

Even though the radical •CH_2_NH_2_ plays a key role in the formation of glycine, its spectrum and mechanism of formation have yet to be directly identified. Bossa et al. reported that the isolation of this radical is difficult because of the reformation of CH_3_NH_2_ after the recombination of •CH_2_NH_2_ with H atom; they consequently employed CO as an H-atom scavenger to diminish the recombination, but observed only formamide and N-methylformamide, not •CH_2_NH_2_, after VUV (vacuum ultraviolet) irradiation of a CH_3_NH_2_/CO binary ice mixture^20^.

Here, we present direct experimental evidence, via IR spectra, of the formation of •CH_2_NH_2_ and CH_2_NH from the reaction of H atom with CH_3_NH_2_ via a H-abstraction tunneling reaction at low temperature, even in darkness. Furthermore, our experimental results showed a tight chemical connection between CH_3_NH_2_ and CH_2_NH through dual H-abstraction and H-addition cycles.

## Results and discussion

To perform H-atom reactions in the laboratory, we co-deposited Cl_2_, CH_3_NH_2_, and *para*-hydrogen (*p*-H_2_) at 3.2 K and irradiated the matrix with light at 365 nm from a light-emitting diode, followed by IR irradiation. The UV photolysis of Cl_2_ at 365 nm generated Cl atom, which is stable toward H_2_ because the reaction Cl + H_2_ → HCl + H is endothermic and has a large barrier^21^. The subsequent IR irradiation excites H_2_ from *ν* = 0 to *ν* = 1 to overcome the energetic limitations so that the Cl atom reacts with H_2_ (*ν* = 1) to form HCl + H. The H atom thus produced reacted with CH_3_NH_2_ during IR irradiation, but the reaction continued even when the matrix was maintained in darkness for a long period because the H atom could migrate slowly in the matrix via tunneling reactions to break and form neighboring H−H bonds (so-called quantum diffusion) to approach CH_3_NH_2_ and react via tunneling reactions. The efficient production of Cl from photolysis of Cl_2_ in a low-temperature matrix requires a diminished cage effect, the production of H requires H_2_ (*ν* = 1), and the migration of H in darkness requires quantum tunneling reaction; all these become possible only in quantum solid *p*-H_2_ having associated unique properties^22,23^.

### Observation of methylamine radical (•CH_2_NH_2_) and methylene imine (CH_2_NH)

**Fig. 1:**
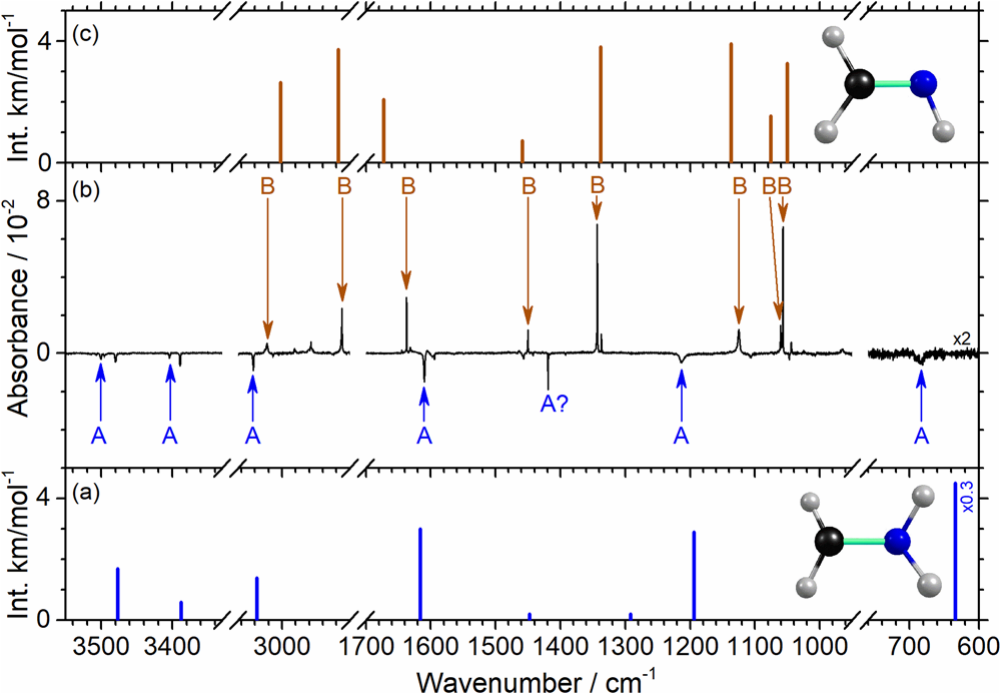
Comparison of observed lines in groups A (•CH_2_NH_2_) and B (CH_2_NH) with theoretical calculations. **a** IR stick spectrum of aminomethyl radical •CH_2_NH_2_. **b** IR difference spectrum after secondary photolysis at 460 nm of a UV/IR- irradiated CH_3_NH_2_/Cl_2_/*p*-H_2_ matrix after being maintained in darkness for 10 h; **c** IR stick spectrum of methylene imine CH_2_NH. Both IR stick spectra in **a** and **c** were simulated according to scaled harmonic vibrational wavenumbers and IR intensities calculated with the B3LYP/aug-cc-pVTZ method.

The IR spectrum of a CH_3_NH_2_/Cl_2_/*p*-H_2_ (1/10/10000) matrix is shown in Supplementary Fig. 1a. The difference spectra after photolysis of the matrix at 365 nm for 30 min, subsequent IR irradiation for 90 min, maintenance of the matrix in darkness for 10 h, and secondary photolysis at 460 nm for 30 min are shown in Supplementary Fig. 1b–e, respectively. New features that appeared after IR irradiation, decreased by ~6% after being in darkness, and decreased by ~30% after secondary photolysis are indicated as group A, whereas those that appeared after IR irradiation, remained nearly constant after being in darkness, and increased by ~90% after secondary photolysis are indicated as group B. The difference spectrum after secondary photolysis at 460 nm (Supplementary Fig. 1e) in representative spectral regions is reproduced in Fig. 1b; lines in group A are pointing downward and those in group B are pointing upward, as indicated with color-coded arrows and labels.

The lines in group A at 3500.5, 3403.6, 3143.3, 3042.6, 1609.9, 1213.6, and 685.5 cm^−1^ agree well, in terms of wavenumbers and relative intensities, with the IR stick spectrum simulated for •CH_2_NH_2_ (Fig. 1a) according to the scaled harmonic vibrational wavenumbers and IR intensities predicted with the B3LYP/aug-cc-pVTZ method; the scaling method is discussed in the Methods section. To understand the perturbations of H_2_ on the IR spectrum of •CH_2_NH_2_, we performed calculations also on •CH_2_NH_2_ surrounded by eighteen H_2_ molecules, either in a hexagonal-closed pack (*hcp*) lattice or randomly (free optimization). The resultant vibrational wavenumbers and IR intensities are compared in Supplementary Table 1 and the simulated IR stick spectra of •CH_2_NH_2_ are presented in Supplementary Fig. 2 to compare with calculations for gaseous •CH_2_NH_2_ and experiments. The perturbation by H_2_ is small (with average absolute deviations 8.8 ± 6.0 and 14.8 ± 8.5 cm^−1^ from the gaseous phase; listed errors represent one standard deviation in fitting) and within calculation errors. This is in line with the fact that observed IR spectra of matrix-isolated species typically showed <1% matrix shifts so that comparison of observed vibrational wavenumbers with predictions of gaseous species was generally performed. The observed lines in group A agree poorly with stick IR spectra of other possible products, as shown in Supplementary Fig. 3.

The symmetric and antisymmetric NH_2_-stretching modes of •CH_2_NH_2_ predicted near 3388 and 3477 cm^−1^ were observed at 3403.6 and 3500.5 cm^−1^, respectively. The symmetric and antisymmetric CH_2_-stretching modes predicted near 3037 and 3134 cm^−1^ were observed at 3042.6 and 3143.3 cm^−1^, respectively. The NH_2_-scissoring mode predicted at 1616 cm^−1^ agrees with the observed feature at 1609.9 cm^−1^. The CN-stretching mode coupled with CH_2_-scissoring, predicted near 1194 cm^−1^, was observed at 1213.6 cm^−1^. The most intense line predicted for the NH_2_-wagging mode at 634 cm^−1^ was observed at 685.5 cm^−1^. Experiments and calculations are compared in Table 1. The observed features in group A can hence be clearly assigned to •CH_2_NH_2_. The average absolute deviation between experiment and prediction is 18.7 ± 15.2 cm^−1^ (1.06 ± 0.8%) for •CH_2_NH_2_. The large deviation for ν_6_ (CH_2_ wag) is typical for this mode because of the inadequacy in describing the double-well potential experienced by N atom, similar to NH_3_. All lines of •CH_2_NH_2_ located in our detection spectral range with predicted IR intensity greater than 6 km mol^−1^ were observed; predicted lines near 1448 and 1292 cm^−1^ have intensity ~2 km mol^−1^ too small to be observed.

**Table 1:** Comparison of observed wavenumbers and relative IR intensities of •CH_2_NH_2_ in solid *p*-H_2_ with their scaled harmonic vibrational wavenumbers and IR intensities predicted with the B3LYP/aug-cc-pVTZ method.

Mode	Sym.	*p-*H_2_		B3LYP/aug-cc-pVTZ			Δν ^e^ (cm^−1^)
		ν/cm^−1^	Intensity^a^ (%)	ν^b^ (cm^−1^)	Intensity (km mol^−1^)	Mode description^c^	
ν_1_	A′	3403.6	20	3388	6	*ν*_*s*_ NH_2_	−15.6
ν_2_	A′	3042.6	45	3037	14	*ν*_*s*_ CH_2_	−5.6
ν_3_	A′	1609.9	100	1616	30	*ρ* NH_2_	6.1
ν_4_	A′	1419.2 (?)	5	1448	2	*δ* CH_2_	—
ν_5_	A′	1213.6	93	1194	29	*ν* CN/*ρ* CH_2_	−19.6
ν_6_	A′	685.5	^d^	634	151	*ω* NH_2_	−51.5
ν_7_	A′	—	—	562	128	*ω* CH_2_	—
ν_8_	A″	3500.5	35	3477	17	*ν*_*a*_ NH_2_	−23.5
ν_9_	A″	3143.3	11	3134	11	*ν*_*a*_ CH_2_	−9.3
ν_10_	A″	—	—	1292	2	*γ* CH_2_/*γ* NH_2_/*ip-def*	—
ν_11_	A″	—	—	913	1	*γ* NH_2_/*γ* CH_2_	—
ν_12_	A″	—	—	432	27	*τ* CH_2_/*τ* NH_2_	—

^a^Integrated intensity relative to the most intense line at 1609.9 cm^−1^ (ν_3_). ^b^Harmonic vibrational wavenumber scaled with the linear equations *y* = (0.9810 ± 0.0126) *x* − (2.9 ± 16.9) and *y* = (0.8907 ± 0.0084) *x* + (233.0 ± 27.2) for regions below and above 2500 cm^−1^, respectively. ^c^Approximate mode description; *ν*: stretch, *ρ*: scissor, *δ*: bend, *γ*: rock, *ω*: wag, *def*: deformation, *τ*: twist, *ip*: in-plane, subscript *a*: antisymmetric, and subscript *s*, symmetric. ^d^Intense absorption of solid *p*-H_2_ near 710 cm^−1^ interfered with the intensity measurement. ^e^ Deviation Δν = ν_calculated_−ν_experimental_.

The lines in group B at 3260.2, 3022.4, 2912.1, 1637.4, 1450.2, 1343.3, 1125.1, 1060.3, and 1056.9 cm^−1^ are readily assigned to methylene imine CH_2_NH, of which the IR spectrum in solid *p*-H_2_ was recorded by Ruzi and Anderson on photodissociation at 193 nm of N–methylformamide in solid *p*-H_2_^24^. The observed vibrational wavenumbers and relative intensities also agree with those predicted for CH_2_NH, as shown in Fig. 1c. A comparison of experiments with theoretical calculations is presented in Supplementary Table 2.

**Fig. 2:**
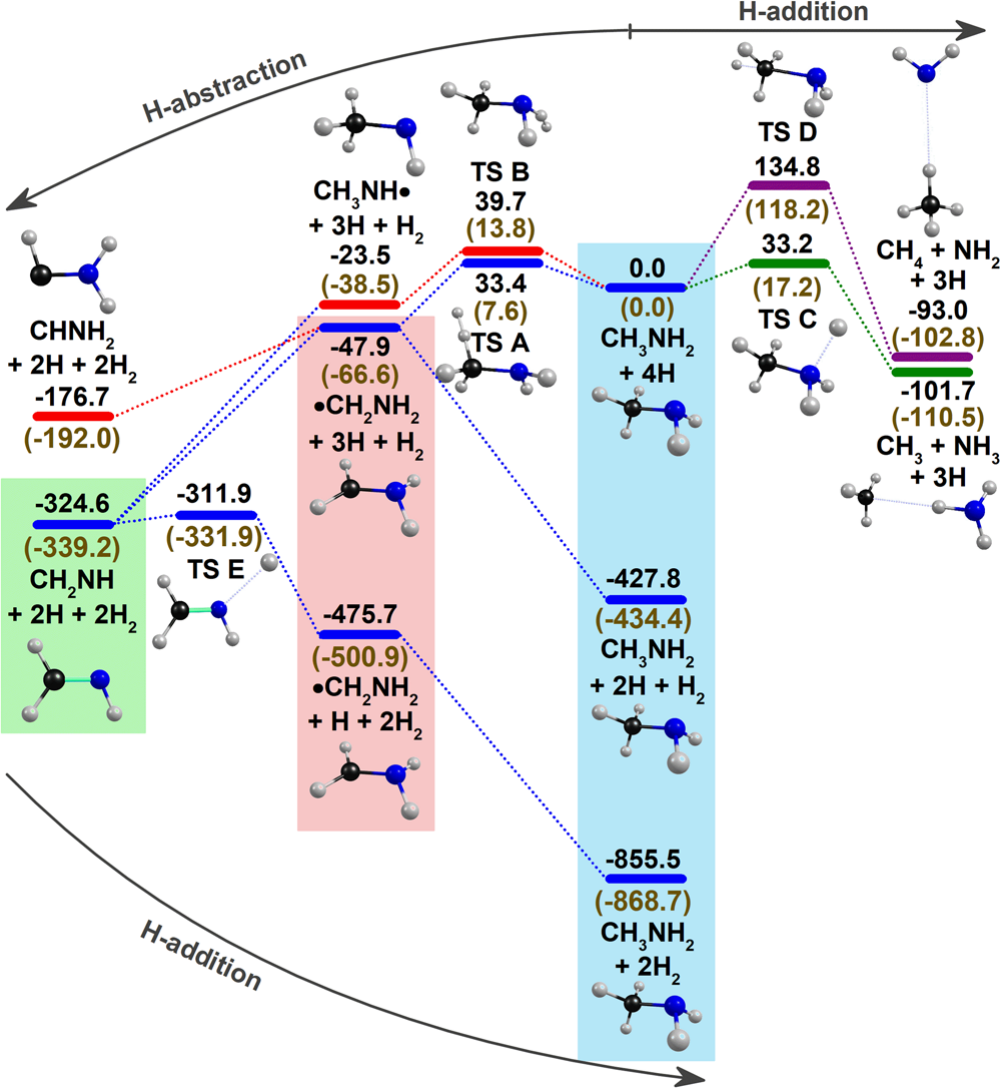
Potential-energy scheme of H-addition and H-abstraction of CH_3_NH_2_ calculated with the CCSD(T)/aug-cc-pVTZ//B3LYP/aug-cc-pVTZ method. Energies corrected for zero-point vibrational energy (ZPVE) are in kJ mol^−1^; those calculated with the B3LYP/aug-cc-pVTZ methods are listed in parentheses for comparison.

To explore the possible products of reaction H + CH_3_NH_2_, we performed quantum-chemical calculations with the CCSD(T)/aug-cc-pVTZ//B3LYP/aug-cc-pVTZ method. The potential-energy scheme for H-abstraction (left side) and H-addition (right side) is shown in Fig. 2; to be self-consistent in terms of energy and species involved, we included all H atoms and H_2_ involved in this reaction network. The first H-abstraction from either moiety CH_3_ or NH_2_ of CH_3_NH_2_ (light blue background) results in the formation of •CH_2_NH_2_ (pink background) or CH_3_NH•, respectively; abstraction on the CH_3_ site has a smaller barrier and is more exothermic. The H-addition to either moiety NH_2_ or CH_3_ of CH_3_NH_2_ results in the rupture of the C−N bond to form CH_3_ + NH_3_ (the most exothermic) or CH_4_ + NH_2_ (involving the largest barrier). Both radicals can proceed with further H-abstraction without barrier to form closed-shell methylene imine CH_2_NH (light green background). The H-addition to •CH_2_NH_2_ to reproduce CH_3_NH_2_ is barrierless, and that to CH_2_NH to form •CH_2_NH_2_ has a small barrier (~13 kJ mol^−1^).

The observation of •CH_2_NH_2_ (group A) and CH_2_NH (group B) agrees with the predicted minimal paths for consecutive H-abstraction of CH_3_NH_2_. The H-abstraction of •CH_2_NH_2_ to form CH_2_NH is barrierless, whereas that of CH_3_NH_2_ to form •CH_2_NH_2_ has barrier ~33 kJ mol^−1^, small enough for the tunneling reaction to occur even at 3.2 K. Our previous observations of H-abstraction of methanol^25^, formamide^26^, methyl formate^27^, acetamide^28^, acetic acid^29^, and glycine^30^ by H atoms showed predicted barriers of 36, 26, 41, 41, 43, and 29 kJ mol^−1^, respectively. Although the calculations showed that H abstraction on the amino hydrogen to form CH_3_NH• has a barrier ~40 kJ mol^−1^, we did not observe CH_3_NH•, which can be readily identified at 3241.5, 2828.9, 2795.5, 1365.8, 1025.2 cm^−1^ in solid *p*-H_2_ as reported by Ruzi and Anderson^24^.

The destruction of •CH_2_NH_2_ to form CH_2_NH upon irradiation at 460 nm also agrees with the TD-B3LYP/aug-cc-pVTZ calculations showing an absorption band near 450 nm corresponding to HOMO (α) → LUMO (α) for •CH_2_NH_2_ (Supplementary Figs. 4 and 5 and Supplementary Table 3), but no absorption for CH_3_NH_2_ at wavelength greater than 250 nm. We observed also the formation of a small proportion of NH_3_ (~8% of •CH_2_NH_2_), indicating that H addition to CH_3_NH_2_ produced CH_3_ + NH_3_. We observed also CH_3_Cl, likely produced from the reaction of CH_3_ with Cl.

### Isotopic Substitution reaction

We performed also experiments on partially deuterated methyl amine, CD_3_NH_2_. The representative spectra at various stages are depicted in Supplementary Fig. 6. The IR difference spectrum on secondary photolysis at 460 nm of the UV/IR-irradiated matrix after being maintained in darkness for 10 h is compared with IR stick spectra predicted for •CD_2_NH_2_ and CD_2_NH in Supplementary Fig. 7. Lines in group A′ match with IR lines predicted for •CD_2_NH_2_. Similarly, lines in group B′ match with those predicted for CD_2_NH. In addition to lines in group A′ and group B′, we observed lines at 2125.2, 996.2, 937.6, 838.7, and 769.7 cm^−1^, denoted group C′, that can be assigned to CD_2_HNH_2_, as presented in Supplementary Fig. 8. This observation confirms that H addition to •CD_2_NH_2_ produced CD_2_HNH_2_. A comparison of experimental results and calculations for •CD_2_NH_2_, CD_2_NH, and CD_2_HNH_2_ is listed in Supplementary Tables 4−6. This isotopic experiment provides not only spectral confirmation of •CH_2_NH_2_ and CH_2_NH, but also direct evidence of the conversion of •CH_2_NH_2_ back to CH_3_NH_2_ by H-addition, consistent with the report by Oba et al. that H−D substitution of solid CH_3_NH_2_ (and its isotopologues) is more rapid in the CH_3_ moiety than in the NH_2_ moiety when CH_3_NH_2_ reacts with H or D atoms under astrophysically relevant conditions^31^. Although we performed no experiment on CD_3_ND_2_ to confirm that H + CD_2_ND → •CD_2_NDH occurred, we expect this reaction to occur because of a small barrier.

### Temporal profiles and reaction mechanism

**Fig. 3:**
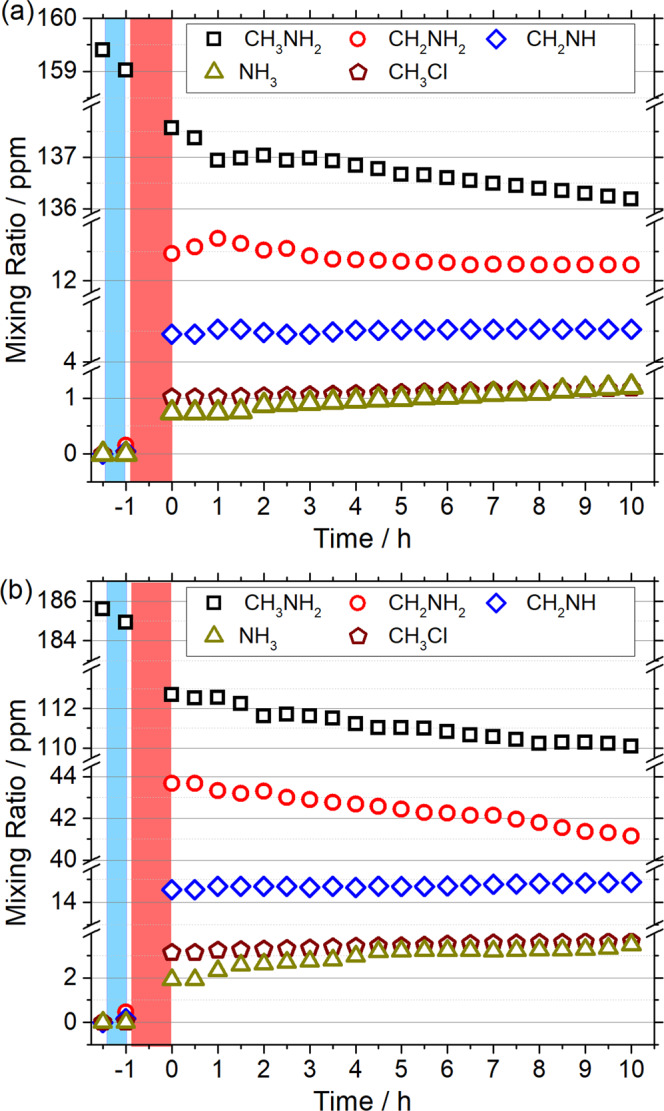
Temporal evolution of mixing ratios of CH_3_NH_2_, •CH_2_NH_2_, CH_2_NH, NH_3_, and CH_3_Cl upon UV and IR irradiation of CH_3_NH_2_/Cl_2_/*p*-H_2_ matrices, followed by maintenance in darkness. **a** [H]_0_/[CH_3_NH_2_]_0_ ≈ 2.1 and [CH_3_NH_2_]_0_ = 159 ppm, **b** [H]_0_/[CH_3_NH_2_]_0_ ≈ 7.2 and [CH_3_NH_2_]_0_ = 186 ppm. The regions shaded with blue and red correspond to periods of UV and IR irradiation, respectively.

The temporal evolution of the mixing ratios of each species is presented in Fig. 3 for two conditions with [H]_0_/[CH_3_NH_2_]_0_ ≈ 2.1 ([CH_3_NH_2_]_0_ ≈ 159 ppm) and [H]_0_/[CH_3_NH_2_]_0_ ≈ 7.2 ([CH_3_NH_2_]_0 _≈ 186 ppm); details are discussed in Supplementary Note 1. •CH_2_NH_2_ was the dominant product, followed by CH_2_NH. In the H-deficient experiment, we observed an initial increase of •CH_2_NH_2_, followed by a decrease to reach a constant mixing ratio, consistent with the two-step mechanism for the formation from the first H abstraction of CH_3_NH_2_ and the destruction due to the second H abstraction; the *anti*-correlation between the mixing ratios of CH_3_NH_2_ and •CH_2_NH_2_ was clearly visible. In the H-rich experiment, •CH_2_NH_2_ and CH_2_NH increased significantly upon IR irradiation, and •CH_2_NH_2_ continuously decreased in darkness, indicating more significant H abstraction of •CH_2_NH_2_ due to the presence of more H atoms.

**Fig. 4:**
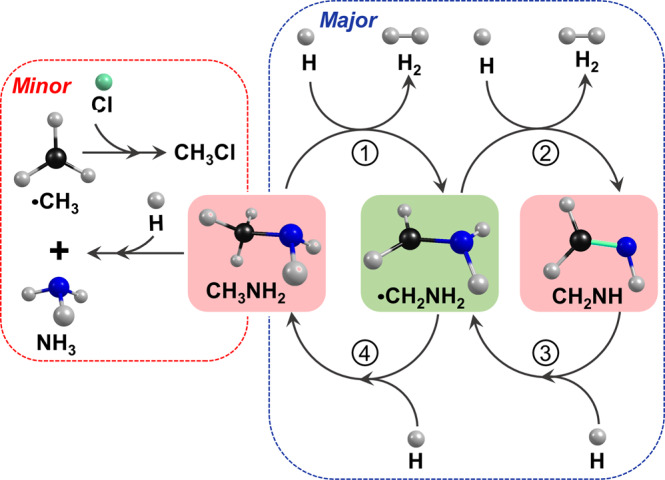
Dual-cycle mechanism of H-abstraction and H-addition reactions connecting CH_3_NH_2_, •CH_2_NH_2_, and CH_2_NH and the formation of CH_3_ and NH_3_. The area on the right with blue dotted boundary represents the major dual-cycle channels connecting CH_3_NH_2_, •CH_2_NH_2_, and CH_2_NH with two sets of H-abstraction and H-addition reactions. The area on the left with red dotted boundary represents minor reactions channels of the decomposition of CH_3_NH_2_ to form CH_3_ and NH_3_ upon H addition).

The observation of consecutive H abstraction of CH_3_NH_2_ to form •CH_2_NH_2_ and CH_2_NH, and their H-addition to reform CH_3_NH_2_ and •CH_2_NH_2_, respectively, connects CH_3_NH_2_, •CH_2_NH_2_, and CH_2_NH via a dual-cycle mechanism shown in Fig. 4, similar to that among formamide HC(O)NH_2_, H_2_NCO and HNCO^26^. The first H-abstraction channel (reaction 1) depicted in Fig. 4 was reported by Garrod^5^ and Suzuki^6^ in their theoretical models. Hydrogenation of solid HCN and CH_2_NH at low temperature conducted by Theule et al.^32^ resulted in the formation of CH_3_NH_2_ directly, indicating the presence of two consecutive H addition (reactions 3 and 4), even though •CH_2_NH_2_ was not observed directly. In the present study, all guest molecules are well isolated in solid *p*-H_2_ at low temperature so that free radicals such as •CH_2_NH_2_ have much better chance to be trapped and maintained. Furthermore, because the hydrogenation experiments by Theule et al.^32^ were carried out by hydrogen bombardment, •CH_2_NH_2_ is expected to react readily with a second hydrogen atom to form the end product CH_3_NH_2_. Our observations of the formation of •CH_2_NH_2_ and CH_2_NH in darkness and the formation of CD_2_HNH_2_ from H reactions with CD_3_NH_2_ further support the dual-cycle mechanism.

We understand that our experimental conditions do not mimic the ISM conditions closely, so our results cannot be applied directly to the reactions in the ISM. For example, in the case of water ice environments, the interaction between water and the guest species might be stronger so that the stability and reactivity of radicals are different from the gaseous phase, as demonstrated by the simulations of radical-radical reactions on icy surfaces by Enrique-Romero et al.^33^ Nevertheless, our results clearly indicate that reaction of H with methylamine CH_3_NH_2_ produces •CH_2_NH_2_, an important radical precursor for the formation of glycine, directly supporting the mechanism, reaction (1), proposed by Ioppolo et al.^19^ for the formation of glycine under conditions similar to dark interstellar clouds with no need for UV irradiation or cosmic rays. The mobility of chemical reactants in the bulk ice is assumed through a swapping mechanism that was supported by laboratory work^34,35^ and theoretical investigations^5^; this mechanism likely brings H atom and CH_3_NH_2_ in close proximity to react.

## Conclusions

The IR spectrum of •CH_2_NH_2_ is previously unreported; it provides a unique tool to probe this important intermediate, a precursor of glycine. We showed also the order of the consecutive H abstraction of CH_3_NH_2_ to be on the CH_3_ moiety first (to produce •CH_2_NH_2_), followed by the NH_2_ moiety (to produce CH_2_NH); the presence of the back reaction (H addition to radicals) was confirmed in experiments of H + CD_3_NH_2_ to produce CD_2_HNH_2_. This dual-cycle mechanism provides an explanation that CH_2_NH and CH_3_NH_2_ might be chemically connected. CH_2_NH was also considered to be a precursor of glycine via reactions with CO + H_2_O or CO_2_ + H_2_ in hot core or cold molecular clouds^36–38^.

## Methods

### Experimental details

A description on the IR absorption matrix-isolation system using *p-*H_2_ as a matrix host is available elsewhere^22,39,40^. A nickel-coated copper plate at 3.2 K served as a cold substrate for matrix samples and also for reflection absorption spectra. A closed-cycle helium refrigerator system was used to cool the substrate. A gaseous mixture of CH_3_NH_2_/Cl_2_/*p*-H_2_ (1/10/10000) was deposited, typically over a period of 9 h at a flow rate ~7 STP cm^3^ min^−1^ (STP indicates standard temperature 273 K and pressure 760 torr). The photolysis of Cl_2_ in the matrix at 365 nm (light-emitting diode, 5 W) produced Cl atoms; subsequent IR irradiation (unfiltered external SiC source) promoted the reaction Cl + H_2_ (*v* = 1) → H + HCl, resulting in the generation of H atoms. The matrix was maintained in darkness for 10 h to study H-tunneling reactions and was further photolyzed with light at 460 nm to distinguish lines of each species. To generate *p*-H_2_, normal H_2_ (99.9999%) was passed through a trap at 77 K before entering a converter containing an iron(III)-oxide catalyst cooled to 12.9 K with a closed-cycle helium refrigerator. Methylamine (CH_3_NH_2,_ Sigma-Aldrich, purity ≥99%), having vapor pressure ~1400 Torr at 293 K, was used to prepare a gaseous mixture CH_3_NH_2_/*p*-H_2_ (1/10000). Methylamine-*d*_3_ (CD_3_NH_2_, Cambridge Isotope Laboratories, ≥98% isotopic purity) was used for isotopic experiments. A Fourier-transform infrared (FTIR) spectrometer equipped with KBr beam splitter and HgCdTe detector at 77 K was used to record IR absorption spectra covering a spectral range 600−4000 cm^−1^. A total of 200 interferometric scans at a resolution of 0.25 cm^−1^ were typically recorded at each stage of the experiment.

### Quantum-chemical calculations

Geometry optimizations and vibrational analyses (wavenumbers and IR intensities) were calculated with the Gaussian16 program package^41^. Density-functional theory calculations were performed using B3LYP functionals^42^ and standard Dunning’s correlation-consistent basis set augmented with diffuse functions, aug-cc-pVTZ^43^. Furthermore, calculations on single-point electronic energies with the method coupled cluster with single and double and perturbative triple excitations, CCSD(T)^44^, were performed on geometries obtained with the B3LYP/aug-cc-pVTZ method; zero-point vibrational energies (ZPVE) were corrected according to the harmonic vibrational wavenumbers calculated with the B3LYP method. Both harmonic and anharmonic vibrational wavenumbers were calculated with the B3LYP/aug-cc-pVTZ method. To obtain scaled harmonic vibrational wavenumbers, plots of observed wavenumbers against calculated harmonic vibrational wavenumbers were employed for two separate regions. Two linear equations, *y* = (0.9810 ± 0.0126) *x* − (2.9 ± 16.9) and *y* = (0.8907 ± 0.0084) *x* + (233.0 ± 27.2), were derived for regions 800−1700 and 2900−3600 cm^−1^, respectively; *y* is the observed wavenumber and *x* is the calculated harmonic vibrational wavenumber. The average absolute deviation is 5.1 ± 3.8 cm^−1^ between experiments and scaled harmonic vibrational wavenumbers of CH_3_NH_2_ and 16.4 ± 22.7 cm^−1^ between experiments and anharmonic vibrational wavenumbers of CH_3_NH_2_. The same equations were employed to scale harmonic vibrational wavenumbers of all species considered in this work.

## Supplementary information

Supplementary Information

Peer Review File

## Data Availability

The data that support the plots within this paper and other findings of this study are available from the corresponding author upon reasonable request.
