# Supplementary material for: A chemical link between methylamine and methylene imine and implications for interstellar glycine formation
Source: Commun Chem. 2022 May 12;5:62. doi: 10.1038/s42004-022-00677-5 (PMC9814145; doi:10.1038/s42004-022-00677-5)
Supplement: Supplementary file 1 — Supplementary Information [file 42004_2022_677_MOESM1_ESM.pdf]

Supplementary Information

A chemical link between methylamine and methylene imine and implications for interstellar glycine formation

Prasad Ramesh Joshi[1] and Yuan-Pern Lee[1,2,3] *

[1] Department of Applied Chemistry and Institute of Molecular Science, National Chiao Tung University, Hsinchu 300093, Taiwan.

[2] Center for Emergent Functional Matter Science, National Chiao Tung University, Hsinchu 300093, Taiwan

[3] Institute of Atomic and Molecular Sciences, Academia Sinica, Taipei 106319, Taiwan

* Email – yplee@nucu.edu.tw (Y.-P.L)

Index

Supplementary Note 1. Estimates of mixing ratios 1

Supplementary Fig. 1. Representative spectra of a $CH_3NH_2/Cl_2/p\text{-}H_2$ (1/10/10000) matrix recorded after various steps of an experiment 3

Supplementary Fig. 2. Comparison of lines in group A with IR stick spectra predicted for $\bullet CH_2NH_2$ in the gaseous and solid phases 4

Supplementary Fig. 3. Comparison of lines in groups A and B with IR stick spectra of predicted H-abstraction and H-addition products, $CH_3NH\bullet$, $CHNH_2$, CH_3 + NH_3, and CH_4 + NH_2 5

Supplementary Fig. 4. UV spectra of CH_3NH_2 (a) and $\bullet CH_2NH_2$ (b) calculated with the TD-B3LYP/aug-cc-pVTZ method 6

Supplementary Fig. 5. The frontier molecular orbital diagram and the isovalue contours (0.03 a.u.) of $\bullet CH_2NH_2$ calculated with the TD-B3LYP/aug-cc-pVTZ method 7

Supplementary Fig. 6. Representative spectra of a $CD_3NH_2/Cl_2/p\text{-}H_2$ (1/10/10000) matrix recorded after various steps of an experiment 8

Supplementary Fig. 7. Comparison of observed lines in groups A′ and B′ with theoretical calculations in experiments with $CD_3NH_2/Cl_2/p\text{-}H_2$ 9

Supplementary Fig. 8. Comparison of lines in groups A′, B′, and C′ with IR stick spectra of predicted H-abstraction and H-addition products, $\bullet CD_2NH_2$, CD_2NH, CD_2HNH_2, $CD_3NH\bullet$, $CDNH_2$, CD_3 + NH_3, and CD_3H + NH_2 10

Supplementary Fig. 9. Temporal evolution of mixing ratios of CD_3NH_2, $\bullet CD_2NH_2$, CD_2NH, CD_2HNH_2, NH_3, and CD_3Cl upon UV and IR irradiation of $CD_3NH_2/Cl_2/p\text{-}H_2$ matrices, followed by maintenance in darkness 11

Supplementary Table 1. Comparison of scaled wavenumbers and relative IR intensities of $\bullet CH_2NH_2$ in the gaseous and solid phases (*hcp* lattice and random) predicted with the B3LYP/aug-cc-pVTZ method 12

Supplementary Table 2. Comparison of experimental vibrational wavenumbers and IR intensities of CH_2NH with those calculated with the B3LYP/aug-cc-pVTZ method 13

Supplementary Table 3. Vertical excitation wavelengths and oscillator strengths of electronic excitations of $\bullet CH_2NH_2$ predicted with the TD-B3LYP/aug-cc-pVTZ method 14

Supplementary Table 4. Comparison of observed wavenumbers and relative IR intensities of $\bullet CD_2NH_2$ in solid $p\text{-}H_2$ with their scaled harmonic vibrational wavenumbers and IR intensities predicted with the B3LYP/aug-cc-pVTZ method 15

Supplementary Table 5. Comparison of experimental vibrational wavenumbers and IR intensities of CD_2NH with those calculated with the B3LYP/aug-cc-pVTZ method 16

Supplementary Table 6. Comparison of observed wavenumbers and relative IR intensities of CD_2HNH_2 in solid $p\text{-}H_2$ with their scaled harmonic vibrational wavenumbers and IR intensities predicted with the B3LYP/aug-cc-pVTZ method 17

Supplementary References 18

Supplementary Note 1. Estimates of mixing ratios

Two parameters, optical path length of the matrix (determined by the IR absorption of p-H_2, as described by Fajardo)[1] and predicted harmonic IR intensities, were employed to estimate mixing ratios of species formed during the reaction H + CH_3NH_2 using the method of Tam and Fajardo.[2] We employed an estimated optical path length 0.9 ± 0.2 mm of the matrix sample and integrated absorbance of lines of CH_3NH_2 (2987.5−2976.6, 1045.6−1041.7, and 803.8−785.6 cm^{-1}), •CH_2NH_2 (3046.1−3039.4, 1618.2−1603.9, and 1223.2−1201.3 cm^{-1}), CH_2NH (3026.7−3019.6, 1346.5−1341.4, and 1058.9−1055.8, cm^{-1}), CH_3Cl (3038.5−3034.9 and 1355.5−1352.4 cm^{-1}), and NH_3 (969.3−966.9 cm^{-1}) to evaluate the mixing ratios; values in parentheses indicate the spectral region of integration. To diminish the possibly large errors (expected to be as much as factor 2) originating from the calculated harmonic IR intensities, several spectral lines of one species were averaged when practical. The initial mixing ratio of hydrogen atom, $[H]_0$, was estimated from the mixing ratio of HCl produced on UV and IR irradiation.

Figure 3 presents mixing ratios of CH_3NH_2, •CH_2NH_2, CH_2NH, CH_3Cl, and NH_3 during the course of an entire experimental period; blue and red shaded regions represent periods of UV and IR irradiation, respectively, and time zero specifies the end of IR irradiation and the beginning of darkness for a matrix at 3.2 K. Mixing ratios for two representative experiments, a H-deficient experiment with $[H]_0/[CH_3NH_2]_0 \approx 2.1$ and $[CH_3NH_2]_0$ = 159 ppm, and a H-rich experiment with $[H]_0/[CH_3NH_2]_0 \approx 7.2$ and $[CH_3NH_2]_0$ = 186 ppm, are shown in traces a and b of Figure 3, respectively. Upon UV photolysis, negligibly small amounts of •CH_2NH_2 and CH_2NH were produced with the destruction of CH_3NH_2 in both experiments; the mixing ratios of •CH_2NH_2 and CH_2NH were less than 1 % relative to those observed after IR irradiation.

In the H-deficient experiment, upon IR irradiation CH_3NH_2 decreased by about 22.6 ppm, whereas •CH_2NH_2 and CH_2NH increased by about 12.4 and 4.4 ppm, respectively, from the H-abstraction channels; CH_3Cl and NH_3 increased by about 1.0 and 0.7 ppm, respectively, presumably from the H-addition channel to form •CH_3 + NH_3. The increased mixing ratios of •CH_2NH_2 and CH_2NH accompanying the decreased mixing ratio of CH_3NH_2 support the formation of these products from a consecutive H-abstraction in H + CH_3NH_2, whereas the observation of NH_3 and CH_3Cl, in which CH_3Cl was likely produced from reaction Cl + •CH_3, indicates the formation of •CH_3 and NH_3 from the H-addition in H + CH_3NH_2. When the matrix was maintained in darkness for 10 h, the mixing ratios of CH_3NH_2 and •CH_2NH_2 decreased by 1.4 and 0.2 ppm, respectively, whereas the mixing ratios of CH_2NH, NH_3 and CH_3Cl increased by 0.1, 0.4, and 0.2 ppm, respectively.

In the H-rich experiment, the mixing ratio of CH_3NH_2 decreased significantly by 72.2 ppm upon IR irradiation, whereas 43.7 and 14.5 ppm, respectively, of •CH_2NH_2 and CH_2NH were produced. In

contrast, only 3.1 and 1.9 ppm of CH_3Cl and NH_3, respectively, were produced. In darkness, the mixing ratio of CH_3NH_2 and $\bullet CH_2NH_2$ decreased by 2.7 and 2.2 ppm, respectively, whereas the mixing ratio of CH_2NH, NH_3, and CH_3Cl increased by 0.3, 1.5, and 0.5 ppm, respectively.

We performed also experiments on H + CD_3NH_2 with an estimated optical path length 0.9 ± 0.2 mm of the matrix sample. The integrated absorbance of lines of CD_3NH_2 (2079.4–2070.7, 923.8–915.2, and 759.9–744.7 cm^{-1}), $\bullet CD_2NH_2$ (2218.2–2212.9, 1612.3–1604.5, and 1257.0–1245.9 cm^{-1}), CD_2NH (2172.8.7–2169.2, 1284.6–1279.7, and 1033.5–1029.9 cm^{-1}), CD_2HNH_2 (939.5–936.3, 843.3–834.9, and 779.4–766.7 cm^{-1}) CD_3Cl (1025.7–1021.8 cm^{-1}), and NH_3 (969.3−966.9 cm^{-1}) to evaluate the mixing ratios; values in parentheses indicate the spectral region of integration.

Supplementary Fig. 9 presents mixing ratios of CD_3NH_2, $\bullet CD_2NH_2$, CD_2NH, CD_2HNH_2, CD_3Cl, and NH_3 during the course of an entire experimental period; blue and red shaded regions represent periods of UV and IR irradiation, respectively. Mixing ratios for two representative experiments, a H-deficient experiment with $[H]_0/[CD_3NH_2]_0 \approx 1.9$ and $[CD_3NH_2]_0 = 165$ ppm, and a H-rich experiment with $[H]_0/[CD_3NH_2]_0 \approx 7.6$ and $[CD_3NH_2]_0 = 188$ ppm, are shown in traces a and b of Supplementary Fig. 9, respectively.

In the H-deficient experiment, upon IR irradiation CD_3NH_2 decreased by about 15.5 ppm, whereas $\bullet CD_2NH_2$ and CD_2NH increased by about 7.0 and 3.4 ppm, respectively; CD_2HNH_2, CD_3Cl, and NH_3 increased by about 1.8, 0.9, and 0.3 ppm, respectively. The increased mixing ratios of $\bullet CD_2NH_2$ and CD_2NH accompanying the decreased mixing ratio of CD_3NH_2 supported the formation of these products from a consecutive H-abstraction in H + CD_3NH_2, whereas the observation of NH_3 and CD_3Cl, in which CD_3Cl was likely produced from reaction Cl + $\bullet CD_3$, indicates the formation of $\bullet CD_3$ and NH_3 from the H-addition in H + CD_3NH_2. The formation of CD_2HNH_2 indicates the presence of the H-addition reaction of H + $\bullet CD_2NH_2$. When the matrix was maintained in darkness for 10 h, the mixing ratios of CD_3NH_2 and $\bullet CD_2NH_2$ decreased by 1.8 and 0.4 ppm, respectively, whereas those of CD_2NH, CD_2HNH_2, NH_3, and CD_3Cl increased by 0.1, 0.3, 0.1, and 0.2 ppm, respectively.

In the H-rich experiment, the mixing ratio of CD_3NH_2 decreased significantly by 56.9 ppm upon IR irradiation, whereas those of $\bullet CD_2NH_2$, CD_2NH, and CD_2HNH_2 increased by 30.9, 10.0, and 7.1 ppm, respectively. In contrast, only 3.0 and 1.6 ppm of CD_3Cl and NH_3, respectively, were produced. In darkness, the mixing ratio of CD_3NH_2 and $\bullet CD_2NH_2$ decreased by 4.4 and 2.0 ppm, respectively, whereas those of CD_2NH, CD_2HNH_2, NH_3, and CD_3Cl increased by 0.2, 2.0, 1.1, and 0.3 ppm, respectively.

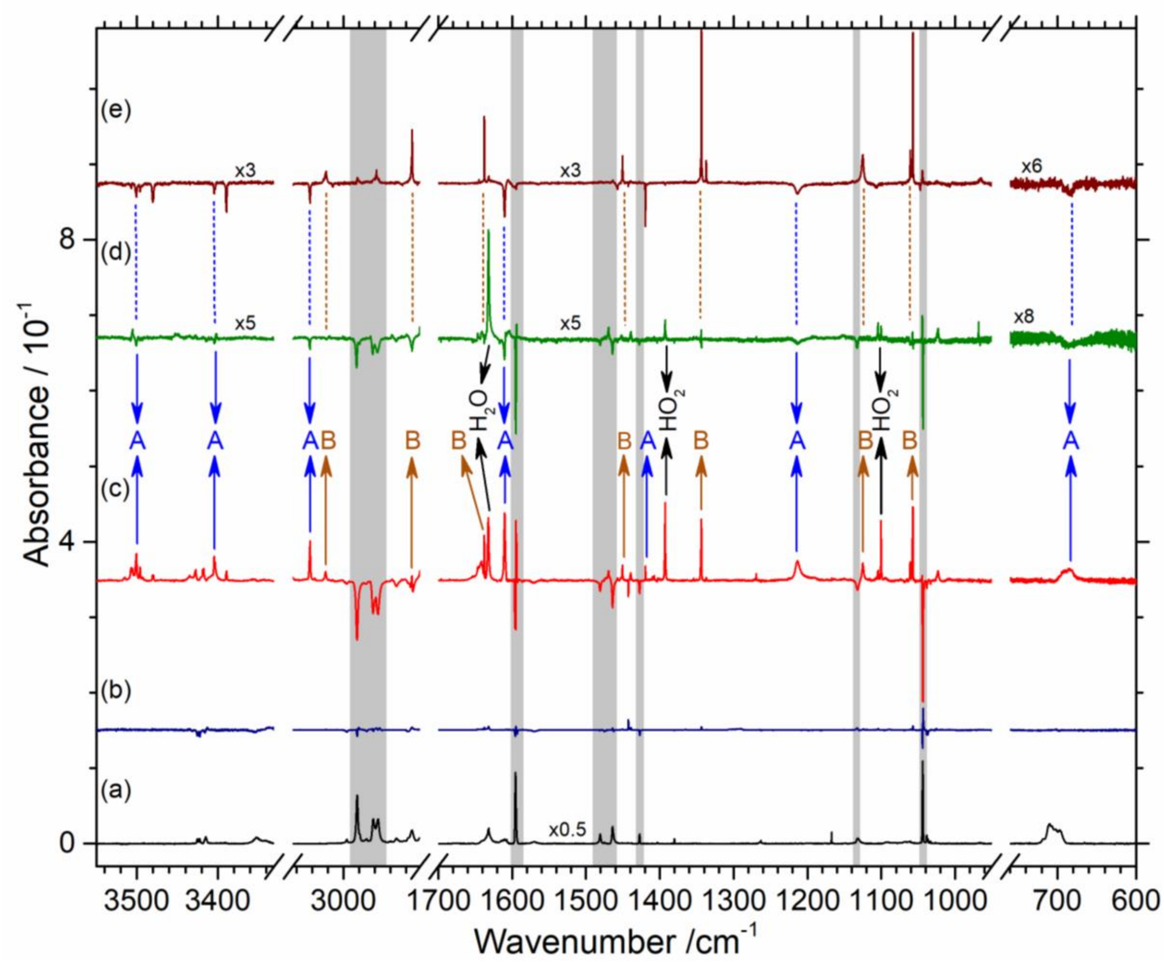

Supplementary Fig. 1 Representative spectra of a $CH_3NH_2/Cl_2/$*p*-H_2 (1/10/10000) matrix recorded after various steps of an experiment. a Spectrum recorded after deposition at 3.2 K for 7 h. **b** Difference spectrum after photolysis of the matrix at 365 nm. **c** Difference spectrum after IR irradiation for 90 min of the UV-irradiated matrix at 365 nm. **d** Difference spectrum after maintaining the matrix in darkness for 10 h. **e** Difference spectrum on secondary photolysis at 460 nm for 30 min. Lines in groups A and B are denoted with blue and brown arrows and labels, respectively; the shaded grey areas indicate spectral regions subject to interference by parent absorption.

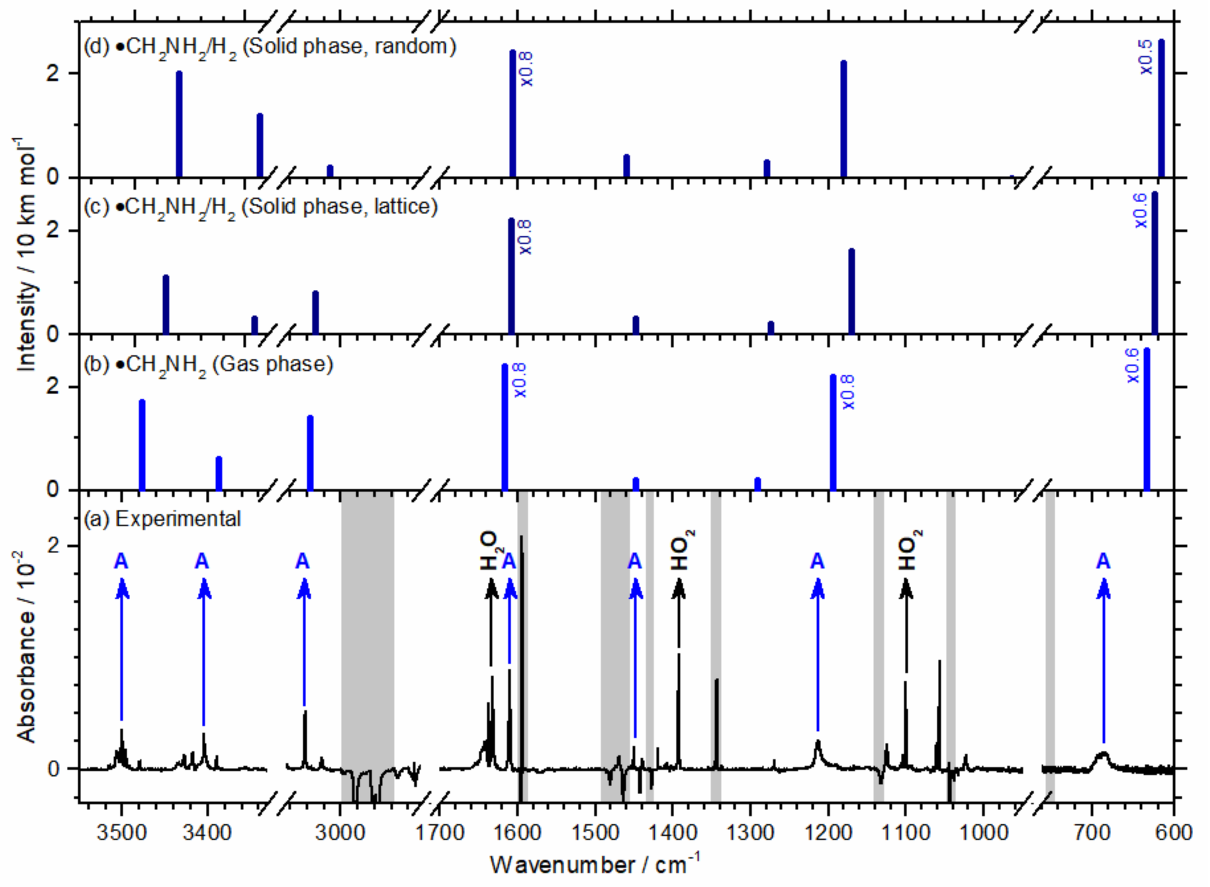

Supplementary Fig. 2 Comparison of lines in group A with IR stick spectra predicted for •CH_2NH_2 in the gaseous and solid phases. a Experimental spectrum taken from Supplementary Fig. 1c; lines in group A are indicated with blue arrows and labels. The stick spectra of **b** •CH_2NH_2 in the gas phase, **c** •CH_2NH_2 in solid phase in which the radical is surrounded by hexagonal-closed packed (hcp) lattice of eighteen H_2 molecules, and **d** •CH_2NH_2 in solid phase in which the radical and the surrounding eighteen H_2 were freely optimized. The harmonic vibrational wavenumbers, calculated with the B3LYP/aug-cc-pVTZ method, were scaled with the same equation as stated in the text. The grey shaded areas indicate the spectral regions subject to the interferences by parent absorption.

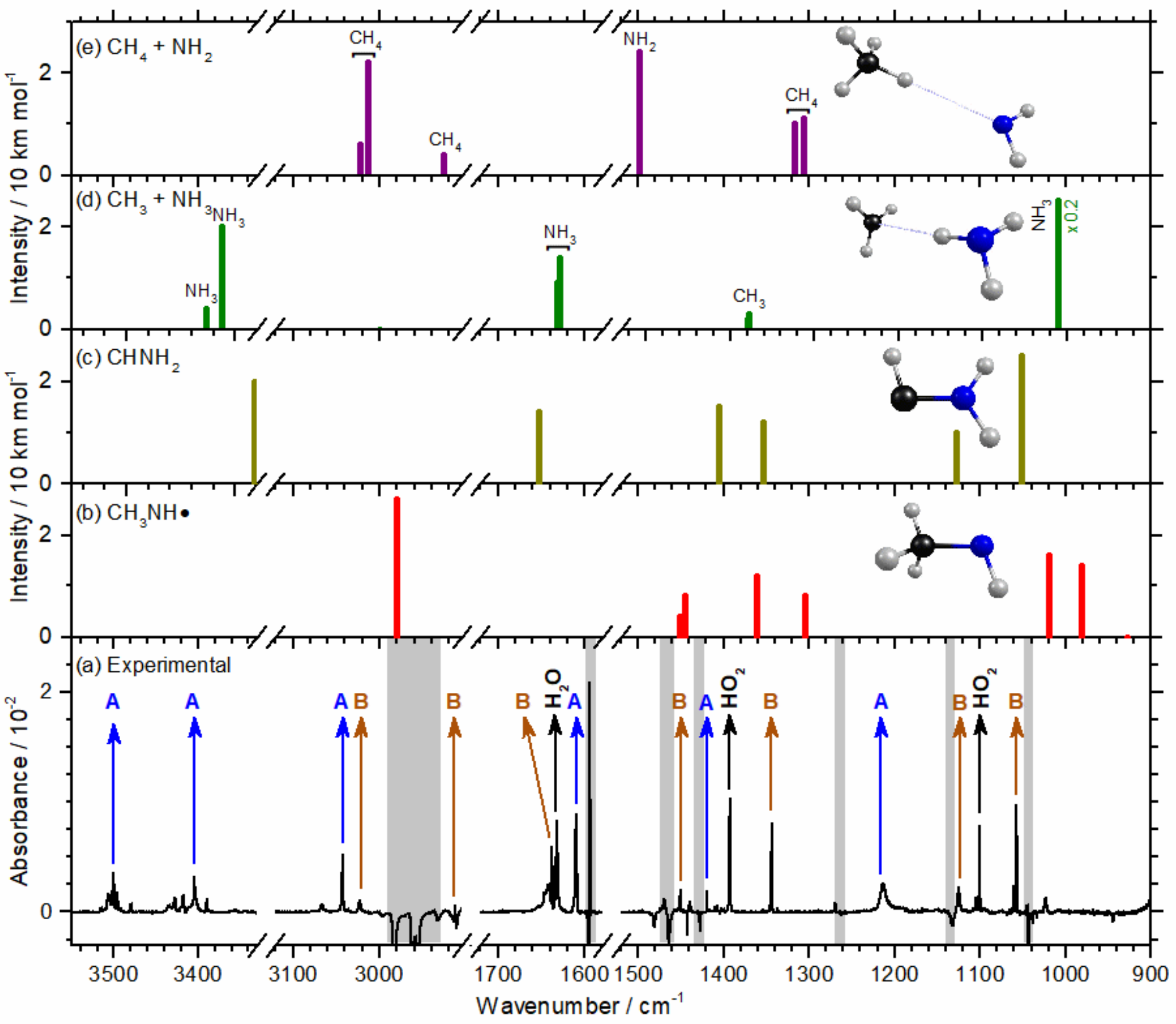

Supplementary Fig. 3 Comparison of lines in groups A and B with IR stick spectra of predicted H-abstraction and H-addition products, CH_3NH•, $CHNH_2$, CH_3 + NH_3, and CH_4 + NH_2. Experimental spectrum is shown in **a**, taken from Supplementary Fig. 1c; lines in group A and B are indicated with blue and orange arrows and labels. The stick spectra of CH_3NH• (**b**), $CHNH_2$ (**c**), CH_3 + NH_3 (**d**), and CH_4 + NH_2 (**e**) follow the scaled harmonic vibrational wavenumber and IR intensities predicted with the B3LYP/aug-cc-pVTZ method. The grey shaded areas indicate the spectral regions subject to the interferences by parent absorption.

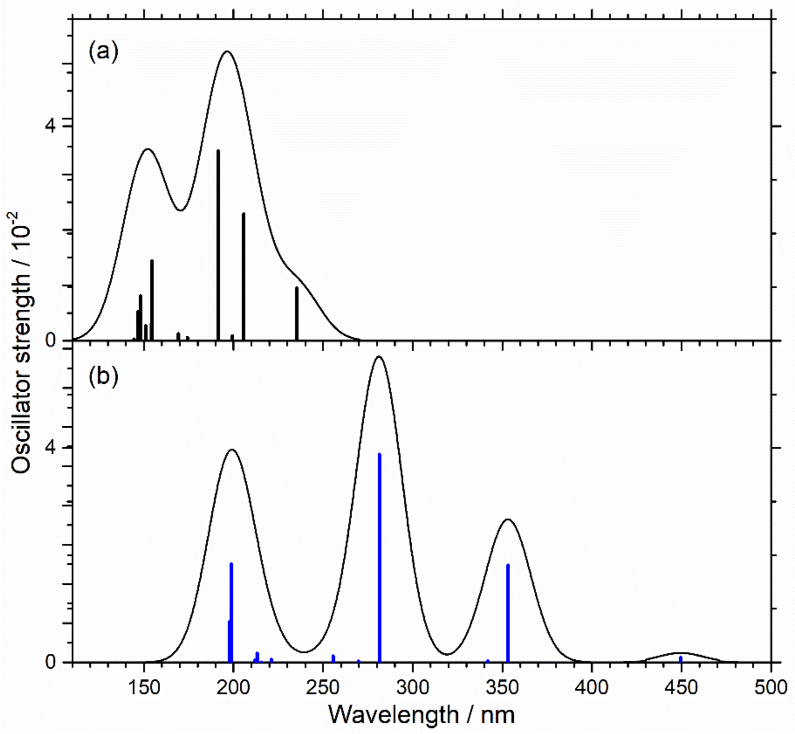

Supplementary Fig. 4 UV spectra of CH_3NH_2 (a) and •CH_2NH_2 (b) calculated with the TD-B3LYP/aug-cc-pVTZ method. The spectra were convoluted with a full width 30 nm at half maximum.

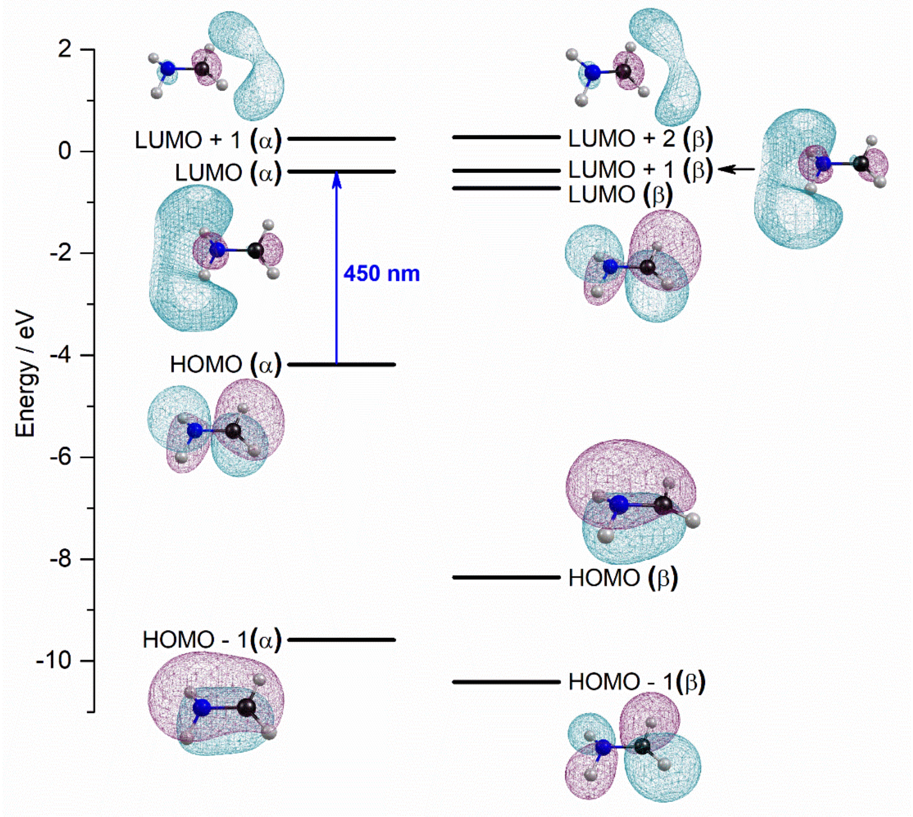

Supplementary Fig. 5 The frontier molecular orbital diagram and the isovalue contours (0.03 a.u.) of •CH_2NH_2 calculated with the TD-B3LYP/aug-cc-pVTZ method.

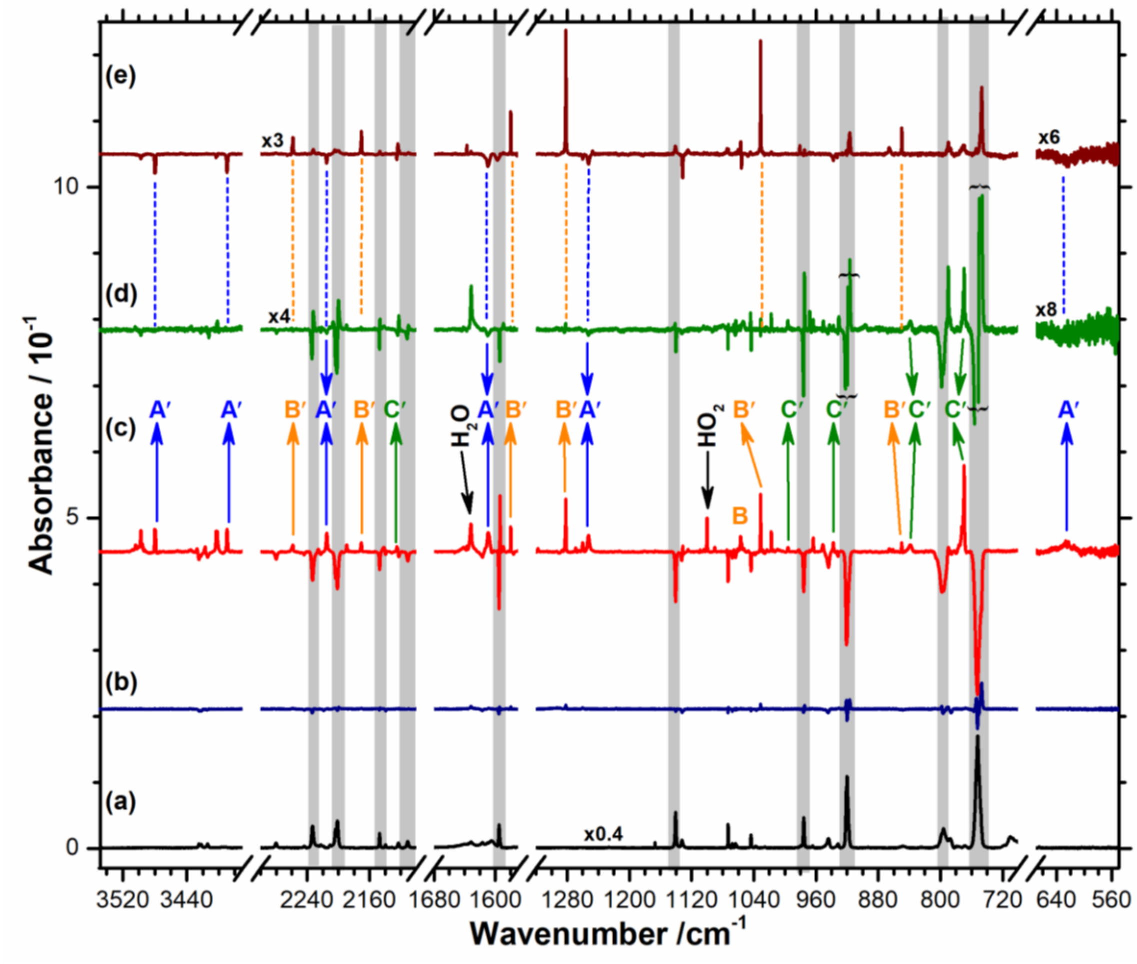

Supplementary Fig. 6 Representative spectra of a $CD_3NH_2/Cl_2/$*p*-H_2 (1/10/10000) matrix recorded after various steps of an experiment. a Spectrum recorded after deposition at 3.2 K for 7 h. **b** Difference spectrum after photolysis of the matrix at 365 nm. **c** Difference spectrum after IR irradiation for 90 min of the UV-irradiated matrix at 365 nm. **d** Difference spectrum after maintaining the matrix in darkness for 10 h. **e** Difference spectrum on secondary photolysis at 460 nm for 30 min. Lines in groups A′, B′ and C′ are denoted with blue, brown, and green arrows and labels, respectively; the shaded grey areas indicate spectral regions subject to interference by parent absorption.

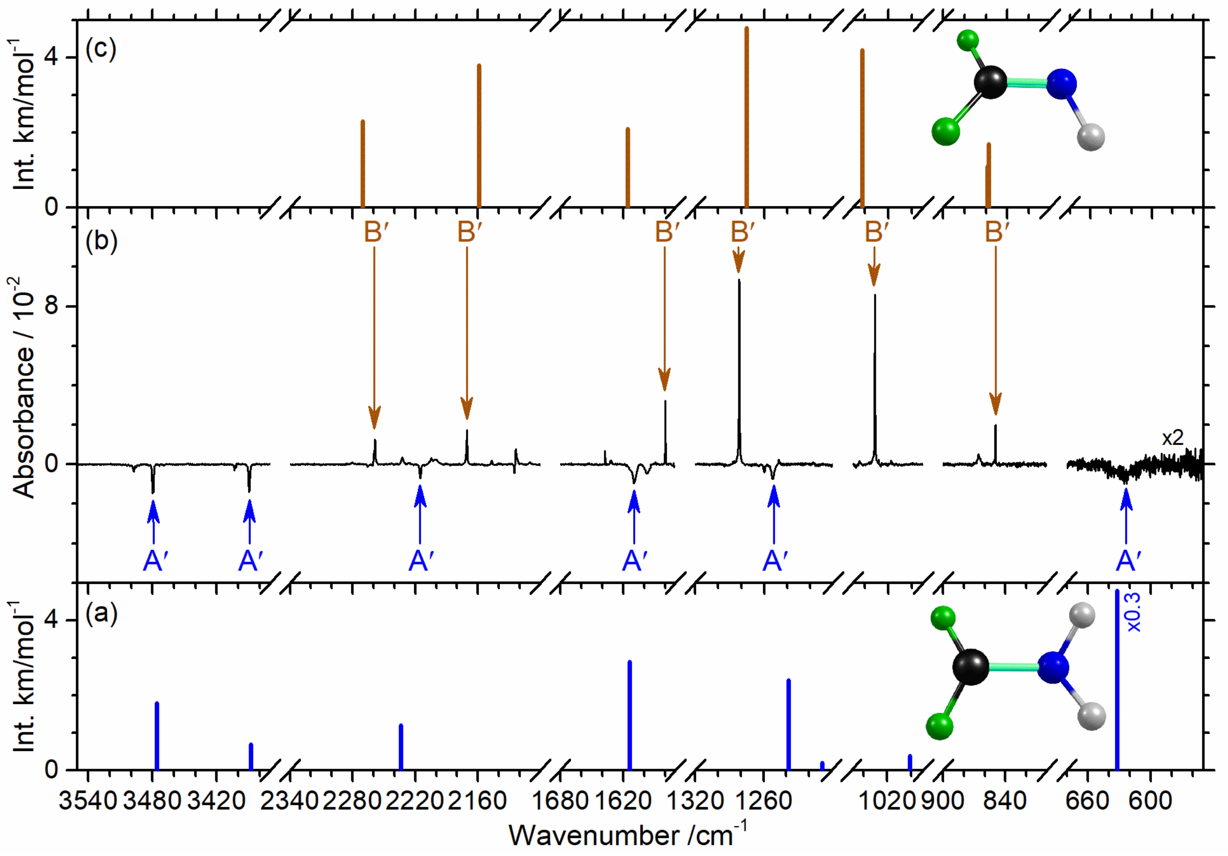

Supplementary Fig. 7 Comparison of observed lines in groups A′ and B′ with theoretical calculations in experiments with $CD_3NH_2/Cl_2/p\text{-}H_2$. **a** IR stick spectrum of deuterated amino-methyl radical $\bullet CD_2NH_2$. **b** IR difference spectrum after secondary photolysis at 465 nm of a UV/IR-irradiated $CD_3NH_2/Cl_2/p\text{-}H_2$ matrix after maintenance in darkness for 10 h. **c** IR stick spectrum of deuterated methylene imine CD_2NH. Both IR stick spectra in traces **a** and **c** were simulated according to scaled harmonic vibrational wavenumbers and IR intensities calculated with the B3LYP/aug-cc-pVTZ method.

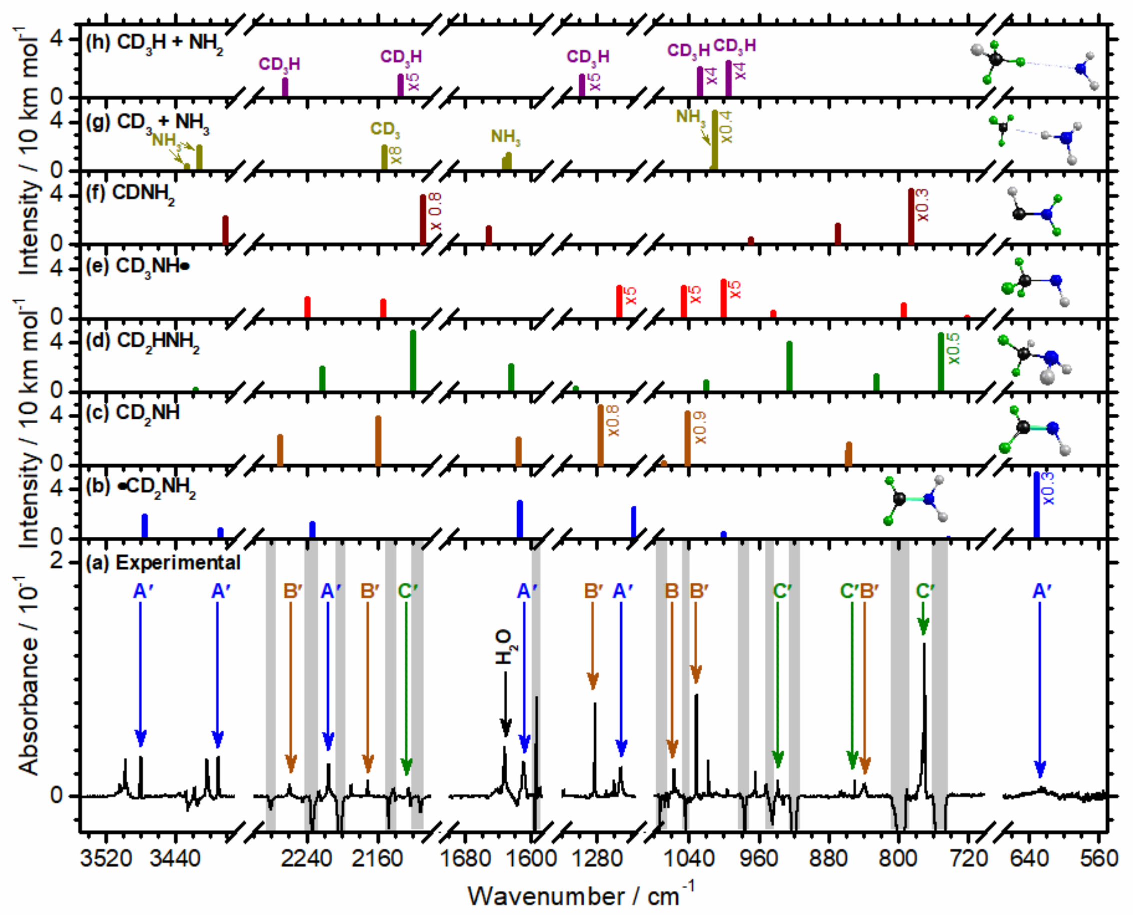

Supplementary Fig. 8 Comparison of lines in groups A′, B′, and C′ with IR stick spectra of predicted H-abstraction and H-addition products, $\bullet CD_2NH_2$, CD_2NH, CD_2HNH_2, $CD_3NH\bullet$, $CDNH_2$, CD_3 + NH_3, and CD_3H + NH_2. Experimental spectrum is shown in **a**, taken from Supplementary Fig. 5c; lines in group A and B are indicated with blue and brown arrows and labels. The stick spectra of $\bullet CD_2NH_2$ (**b**), $\bullet CD_2NH$ (**c**), CD_2HNH_2 (**d**), $CD_3NH\bullet$ (**e**), $CDNH_2$ (**f**), CD_3 + NH_3 (**g**), and CD_3H + NH_2 (**h**) follow the scaled harmonic vibrational wavenumber and IR intensities predicted with the B3LYP/aug-cc-pVTZ method. The grey shaded areas indicate the spectral regions subject to the interferences by parent absorption.

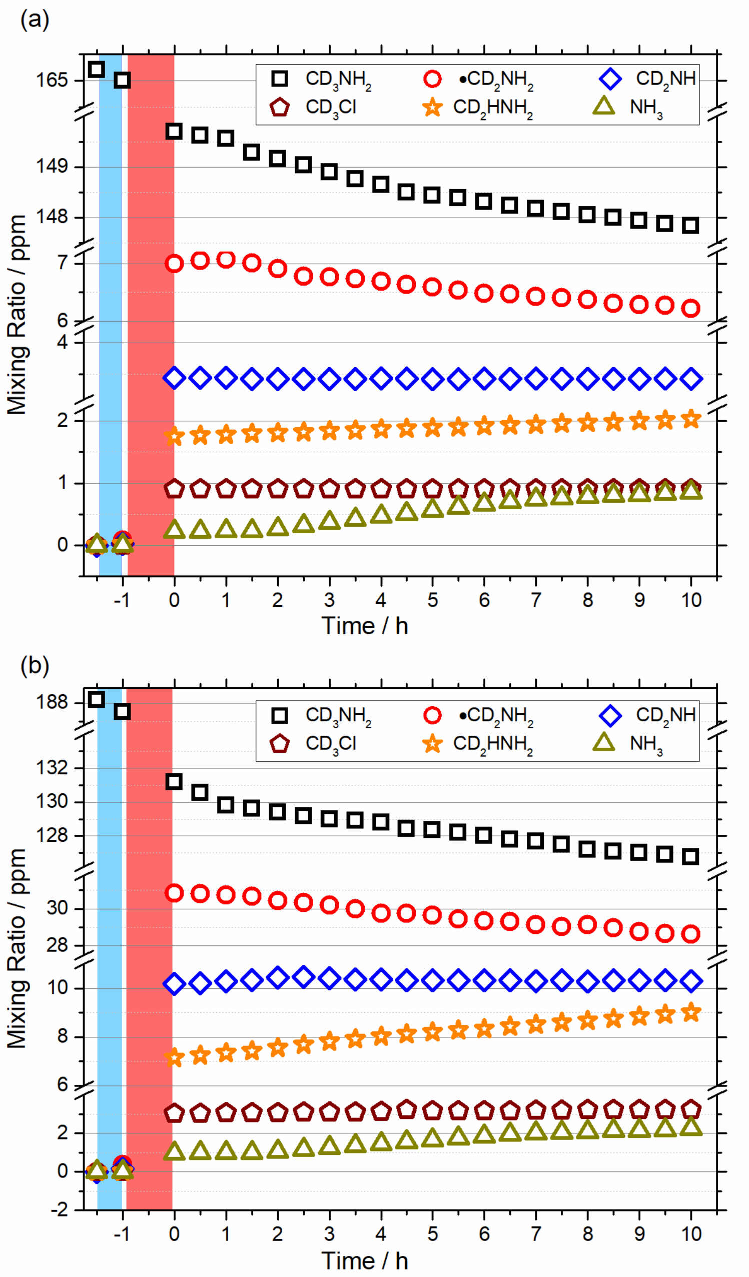

Supplementary Fig. 9 Temporal evolution of mixing ratios of CD_3NH_2, •CD_2NH_2, CD_2NH, CD_2HNH_2, NH_3, and CD_3Cl upon UV and IR irradiation of $CD_3NH_2/Cl_2/$*p*-H_2 matrices, followed by maintenance in darkness. a $[H]_0/[CD_3NH_2]_0 \approx 1.9$ and $[CD_3NH_2]_0 = 165$ ppm. **b** $[H]_0/[CD_3NH_2]_0 \approx 7.6$ and $[CD_3NH_2]_0 = 188$ ppm. The regions shaded with blue and red correspond to periods of UV and IR irradiation, respectively.

Supplementary Table 1 Comparison of scaled wavenumbers and relative IR intensities of •CH_2NH_2 in the gaseous and solid phases (*hcp* lattice and random) predicted with the B3LYP/aug-cc-pVTZ method.

Mode	Sym.	Gas Phase		Solid Phase				Mode description[b]
				hcp lattice		Random		
		ν[a] /cm^{-1}	Intensity /%	ν[a] /cm^{-1}	Intensity /%	ν[a] /cm^{-1}	Intensity /km mol^{-1}	
ν_1	A′	3388	6	3392	4	3387	11	ν_s NH_2
ν_2	A′	3037	14	3030	10	3013	4	ν_s CH_2
ν_3	A′	1616	30	1608	25	1609	36	ρ NH_2
ν_4	A′	1448	2	1447	3	1461	4	δ CH_2
ν_5	A′	1194	29	1171	24	1180	22	ν CN/ρ CH_2
ν_6	A′	634	151	625	126	616	113	ω NH_2
ν_7	A′	562	128	554	118	565	113	ω CH_2
ν_8	A″	3477	17	3472	12	3460	20	ν_a NH_2
ν_9	A″	3134	11	3127	9	3104	13	ν_a CH_2
ν_{10}	A″	1292	2	1274	1	1280	3	γ CH_2/γ NH_2/*ip-def*
ν_{11}	A″	913	1	904	0	935	1	γ NH_2/γ CH_2
ν_{12}	A″	432	27	425	19	415	25	τ CH_2/τ NH_2

[a]Harmonic vibrational wavenumber scaled with the linear equations $y = (0.9810 \pm 0.0126)\, x - (2.9 \pm 16.9)$ and $y = (0.8907 \pm 0.0084)\, x + (233.0 \pm 27.2)$ for regions below and above 2500 cm^{-1}, respectively. [b]Approximate mode description; ν: stretch, ρ: scissor, δ: bend, γ: rock, ω: wag, *def*: deformation, τ: twist, *ip*: in-plane, subscript *a*: anti-symmetric, and subscript *s*, symmetric.

Supplementary Table 2 Comparison of experimental vibrational wavenumbers and IR intensities of CH_2NH with those calculated with the B3LYP/aug-cc-pVTZ method.

		B3LYP/aug-cc-pVTZ			*p*-H_2[c]		Ar[e]	*p*-H_2[f]	Gas[g,h]
mode	sym.	ν[a] /cm^{-1}	int. /km mol^{-1}	ν[b] /cm^{-1}	ν /cm^{-1}	int.[d] /%	ν /cm^{-1}	ν /cm^{-1}	ν /cm^{-1}
ν_1	A′	3290	1	3260	3260.2	5		3260.1	3262.6
ν_2	A′	3002	29	2942	3022.4	28	3035	3026.0	3024.4
ν_3	A′	2917	51	2849	2912.4	54[i]	2921	2912.0	2914.2
ν_4	A′	1673	23	1674	1637.4	27	1639	1637.3	1638.3
ν_5	A′	1459	8	1461	1450.2	16	1452	1450.1	1452.0
ν_6	A′	1338	43	1331	1343.3	100	1347	1343.3	1344.3
ν_7	A′	1076	35	1084	1056.9	92		1057.0	1058.2
ν_8	A″	1137	42	1133	1125.1	51[i]		1124.9	1127.0
ν_9	A″	1050	36	1057	1060.3	26	1061	1060.5	1060.8

[a]Harmonic vibrational wavenumber scaled with linear equations y = (0.9810 ± 0.0126) x − (2.9 ± 16.9) and y = (0.8907 ± 0.0084) x + (233.0 ± 27.2) for values smaller and greater than 2500 cm^{-1}, respectively; see text. [b]Anharmonic vibrational wavenumber. [c]This work. [d]Percentage IR intensities relative to the most intense line at 1343.3 cm^{-1} (ν_6). [e] Quinto-Hernandez, A., Wodtke, A. M., Bennett, C. J., Kim, Y. S. & Kaiser, R. I., *J. Phys. Chem. A* **115**, 250−264 (2011). [f] Ruzi, M. & Anderson, D. T. *J. Chem. Phys.* **137**, 194313 (2012**).** [g]Halonen, L. & Duxbury, G. *Chem. Phys. Lett.* **118**, 246−251 (1985). [h]Halonen, L. & Duxbury, G. *J. Chem. Phys.* **83**, 2091−2096 (1985). [i] Interference of the intense absorptions of CH_3NH_2.

Supplementary Table 3 Vertical excitation wavelengths and oscillator strengths of electronic excitations of •CH_2NH_2 predicted with the TD-B3LYP/aug-cc-pVTZ method.

Excitation state	Wavelength / nm	Oscillator strength f	Assignment
1	449.5	0.0009	HOMO(α) → LUMO(α)
2	353.2	0.0181	HOMO(α) → LUMO + 1(α)
3	341.8	0.0002	HOMO(α) → LUMO + 2(α)
4	281.3	0.0387	HOMO(α) → LUMO + 4(α)
5	269.6	0.0002	HOMO(α) → LUMO + 3(α)
6	255.5	0.0012	HOMO(α) → LUMO + 5(α)
7	221.0	0.0005	HOMO(α) → LUMO + 6(α)
8	215.5	0.0000	HOMO(α) → LUMO + 7(α)
9	213.2	0.0017	HOMO(α) → LUMO + 8(α)
10	212.1	0.0004	HOMO(α) → LUMO + 9(α)
11	198.8	0.0183	HOMO(α) → LUMO + 11(α)
12	197.7	0.0076	HOMO(α) → LUMO + 10(α)

Supplementary Table 4 Comparison of observed wavenumbers and relative IR intensities of •CD_2NH_2 in solid *p*-H_2 with their scaled harmonic vibrational wavenumbers and IR intensities predicted with the B3LYP/aug-cc-pVTZ method.

Mode	Sym.	*p*-H_2			B3LYP/aug-cc-pVTZ			
		ν /cm^{-1}	Intensity[a] /%	D/H ratio	ν[b] /cm^{-1}	Intensity	D/H ratio[c]	Mode Description[d]
ν_1	A′	3389.8	21	0.9956	3387	7	1.0000	ν_s NH_2
ν_2	A′	2215.1	35	0.7280	2234	12	0.7456	ν_s CD_2
ν_3	A′	1607.9	100	0.9987	1614	29	0.9988	ρ NH_2
ν_4	A′	1252.6	80	1.0321	1236	24	1.0352	ν CN/ρ CD_2
ν_5	A′				1001	4	0.6913	δ CD_2
ν_6	A′	625.3	[e]	0.9121	632	185	0.9968	ω NH_2
ν_7	A′				442	69	0.7865	ω CD_2
ν_8	A″	3479.8	31	0.9941	3476	18	0.9997	ν_a NH_2
ν_9	A″	2375.6	18	0.7558	2384	6	0.7607	ν_a CD_2
ν_{10}	A″				1208	2	0.9350	γ CD_2/γ NH_2/*ip-def*
ν_{11}	A″				743	1	0.8140	γ NH_2/γ CD_2
ν_{12}	A″				379	25	0.8773	τ CD_2/τ NH_2

[a]Integrated intensity relative to the most intense line at 1607.9 cm^{-1} (ν_3). [b]Harmonic vibrational wavenumber scaled with the linear equations $y = (0.9810 \pm 0.0126)\, x - (2.9 \pm 16.9)$ and $y = (0.8907 \pm 0.0084)\, x + (233.0 \pm 27.2)$ for regions below and above 2500 cm^{-1}, respectively. [c]Calculated from unscaled harmonic vibrational wavenumbers. [d]Approximate mode description; ν: stretch, ρ: scissor, δ: bend γ: rock, ω: wag, *def*: deformation, τ: twist, *ip*: in-plane, subscript *a*: anti-symmetric, and subscript *s*, symmetric. [e]Intensity measurement was unavailable because of poor ratio of signal to noise in this region.

Supplementary Table 5 Comparison of experimental vibrational wavenumbers and IR intensities of CD_2NH with those calculated with the B3LYP/aug-cc-pVTZ method.

Mode	Sym.	p-H_2			B3LYP/aug-cc-pVTZ				
		ν /cm^{-1}	Int.[a] /%	D/H ratio	ν^b /cm^{-1}	Int. /km mol^{-1}	ν^c /cm^{-1}	D/H Ratio	Mode description[d]
ν_1	A′				3290	1	3257	1.0000	ν NH_2
ν_2	A′	2258.6	35	0.7473	2270	23	2223	0.7562	ν_a CD_2
ν_3	A′	2170.6	22	0.7453	2159	38	2106	0.7401	ν_s CD_2
ν_4	A′	1637.3	[e]	0.9998	1616	21	1621	0.9659	ν C=N
ν_5	A′	1281.8	100	0.8839	1275	60	1268	0.8739	δ CNH
ν_6	A′				1069	2	1076	0.7989	δ CD_2
ν_7	A′	849.7	13	0.8039	858	11	868	0.7974	γ CD_2/ δ HNC
ν_8	A″	1031.3	75	0.9166	1042	47	1040	0.9164	ω CD_2
ν_9	A″	849.7	13	0.8014	857	17	868	0.8161	ρ CD_2

[a]Integrated intensity relative to the most intense line at 1281.8 cm^{-1} (ν_5). [b]Harmonic vibrational wavenumber scaled with the linear equations y = (0.9810 ± 0.0126) x - (2.9 ± 16.9) and y = (0.8907 ± 0.0084) x + (233.0 ± 27.2) for regions below and above 2500 cm^{-1}, respectively. [c]Anharmonic vibrational wavenumber. [d]Approximate mode description; ν: stretch, δ: bend, ρ: rock, γ: rock, ω: wag, subscript a: anti-symmetric, and subscript s, symmetric. [e]Intense absorption of H_2O Interfered with the measurement.

Supplementary Table 6 Comparison of observed wavenumbers and relative IR intensities of CD_2HNH_2 in solid *p*-H_2 with their scaled harmonic vibrational wavenumbers and IR intensities predicted with the B3LYP/aug-cc-pVTZ method.

mode	sym.	*p*-H_2		B3LYP/aug-cc-pVTZ		
		ν /cm^{-1}	Intensity[a] /%	ν[b] /cm^{-1}	intensity /km mol^{-1}	mode description[c]
ν_1	A′			3350	1	ν_s NH_2
ν_2	A′			2969	30	ν CH
ν_3	A′	2125.2	53	2120	49	ν_a CD_2
ν_4	A′	[d]		1625	21	δ NH_2
ν_5	A′			1304	3	CD_2H s-*def*
ν_6	A′			1100	10	δ CD_2/δ CNH
ν_7	A′	996.2	38	1021	35	δ CD_2
ν_8	A′	937.6	41	925	39	ν CN
ν_9	A′	769.7	100	752	93	ω NH_2
ν_{10}	A″			3417	2	ν_a NH_2
ν_{11}	A″	[e]		2223	19	ν_a CD_2
ν_{12}	A″			1355	1	γ NH_2 /δ NCH
ν_{13}	A″			1215	1	γ NH_2 /δ CD_2H
ν_{14}	A″	838.7	15	825	13	γ CD_2H / τ NH_2
ν_{15}	A″			263	35	γ NH_2

[a]Integrated intensity relative to the most intense line at 769.7 cm^{-1} (ν_9). [b]Harmonic vibrational wavenumber scaled with the linear equations $y = (0.9810 \pm 0.0126)\,x - (2.9 \pm 16.9)$ and $y = (0.8907 \pm 0.0084)\,x + (233.0 \pm 27.2)$ for regions below and above 2500 cm^{-1}, respectively. [c]Approximate mode description; ν: stretch, δ: bend γ: rock, ω: wag, *def*: deformation, τ: twist, subscript *a*: antisymmetric, and subscript *s*, symmetric. [d]An intense absorption line of CH_3NH_2 interfered with the intensity measurement. [e]An intense absorption line of CD_3NH_2 interfered with the intensity measurement.

Supplementary References

[1] Fajardo, M. E. *Physics and Chemistry at Low Temperatures; Khriachtchev, L. (Ed.),* Pan Stanford Publishing, Singapore, 187 (2011).

[2] Tam, S. & Fajardo, M. Single and double infrared transitions in rapid-vapor-deposited parahydrogen solids: application to sample thickness determination and quantitative infrared absorption spectroscopy. *Appl. Spectrosc.* **55**, 1634–1644 (2001).
